# Essential Function of the Serine Hydroxymethyl Transferase (SHMT) Gene During Rapid Syncytial Cell Cycles in *Drosophila*

**DOI:** 10.1534/g3.117.043133

**Published:** 2017-05-17

**Authors:** Franziska Winkler, Maria Kriebel, Michaela Clever, Stephanie Gröning, Jörg Großhans

**Affiliations:** Institute for Developmental Biochemistry, Medical School, University of Göttingen, 37077, Germany

**Keywords:** *Drosophila*, serine hydroxymethyl transferase, midblastula transition, metabolism, cell cycle

## Abstract

Many metabolic enzymes are evolutionarily highly conserved and serve a central function in the catabolism and anabolism of cells. The serine hydroxymethyl transferase (SHMT) catalyzing the conversion of serine and glycine and vice versa feeds into tetrahydrofolate (THF)-mediated C1 metabolism. We identified a *Drosophila* mutation in *SHMT* (CG3011) in a screen for blastoderm mutants. Embryos from *SHMT* mutant germline clones specifically arrest the cell cycle in interphase 13 at the time of the midblastula transition (MBT) and prior to cellularization. The phenotype is due to a loss of enzymatic activity as it cannot be rescued by an allele with a point mutation in the catalytic center but by an allele based on the *SHMT* coding sequence from *Escherichia coli*. The onset of zygotic gene expression and degradation of maternal RNAs in *SHMT* mutant embryos are largely similar to that in wild-type embryos. The specific timing of the defects in *SHMT* mutants indicates that at least one of the SHMT-dependent metabolites becomes limiting in interphase 13, if it is not produced by the embryo. Our data suggest that mutant eggs contain maternally-provided and SHMT-dependent metabolites in amounts that suffice for early development until interphase 13.

Genes encoding enzymes mediating basic metabolic pathways such as glycolysis or the synthesis of central metabolites such as purines or pyrimidines are assumed to have cell-essential functions. Due to the diverse but central functions of the corresponding metabolites, mutants of such genes are assumed to have pleiotropic phenotypes. A genetic analysis of these enzymes has not been in the focus of developmental genetics.

The evolutionarily highly conserved SHMT is a pyridoxal phosphate-containing enzyme that converts serine into glycine and vice versa (Figure 1). The released hydroxymethyl (C1) unit is transferred to THF to give rise to N5, N10 methylene THF. THF is the carrier of C1 units and is needed for the synthesis of purines, thymine nucleotides, and S-adenosyl methionine (SAM), the methyl donor of many methylation reactions. Beside the SHMT reaction, C1 units are generated by the cleavage of glycine catalyzed by glycine decarboxylase (GLDC) and by the degradation of histidine.

**Figure 1:**
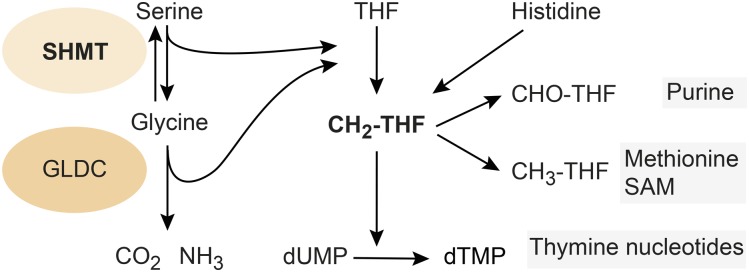
Brief schematic overview of SHMT function and C1 metabolism. SHMT, GLDC, and degradation of histidine feed into the pool of C1 units bound by THF. Metabolites depending on THF-C1 are marked by gray boxes. dTMP, deoxythymidine monophosphate; dUMP, deoxyuridine monophosphate; GLDC, glycine decarboxylase; SAM, S-adenosyl methionine; SHMT, serine hydroxymethyl transferase; THF, tetrahydrofalate.

Mammalian genomes contain two *SHMT* genes, one encoding a cytoplasmic isoform and one encoding a mitochondrial isoform (Andersen and Stover 2009). The single *Drosophila SHMT* gene encodes alternative transcripts, giving rise to a cytoplasmic isoform and mitochondrial isoform proteolytically derived from a longer precursor with a putative N-terminal presequence mediating mitochondrial import. Although the enzymatic function of SHMT is well defined, the genetics of *SHMT* in the context of development has not been investigated in animals so far. An indication for cell-specific functions is provided by the observation that increased expression of *SHMT* or *GLDC* is associated with certain types of tumors (Zhang *et al.* 2012; Kim *et al.* 2015). *GLDC* overexpression was found to be important for tumorigenesis in tumor initiating cells of nonsmall cell lung cancer (Zhang *et al.* 2012). Highly expressed *SHMT2* provides an advantage to tumor cells in poorly vascularized tumor regions (Kim *et al.* 2015).

The early development of embryos in species with large eggs starts under the control of factors (*e.g.*, RNA and proteins) provided by the female. In this period under maternal control, the zygotic genotype has no influence, as zygotic transcription is activated only after a lag phase [for a review concerning *Drosophila* see Foe *et al.* (1993)]. The activation of the zygotic genome coincides with and is required for the MBT, which refers to a switch in development that is visible as changes in morphology and the cell cycle. In *Drosophila*, MBT occurs at ∼2 hr past fertilization after invariantly 13 rounds of nuclear divisions (Foe *et al.* 1993). Following mitosis 13, the cell cycle switches its mode including an extended S phase and a pause in G2 phase. At the same time, the syncytial blastoderm transforms into a cellular blastoderm in a process called cellularization. The mechanisms for timing of zygotic genome activation and MBT, and the underlying functional relationships of the associated processes, are not clear and are a matter of current research (Farrell and O’Farrell 2014; Blythe and Wieschaus 2015a; Harrison and Eisen 2015; Liu and Grosshans 2017).

Here, we isolate a *SHMT* mutation in *Drosophila* by its specific developmental phenotype. Embryos from *SHMT* mutant germline clones develop until MBT and arrest the cell cycle in interphase 13. To initiate a developmental and genetic analysis of *SHMT*, we report here a detailed phenotypic analysis of *SHMT* mutants.

## Materials and Methods

### Genetics: transgenes

The *w* X238 *f* Frt[18E] hs-Flp[122] chromosome was isolated in a screen for mutations with early embryonic phenotypes in germline clones (Vogt *et al.* 2006). The original X238 chromosome was cleaned from a male-sterile mutation by meiotic recombination resulting in the *ypn* X238 *sn* Frt[19A] chromosome. Both chromosomes showed a similar phenotype in germline clones and were rescued with the genomic *SHMT*[+] transgene. Fly stocks were obtained from the Bloomington *Drosophila* Stock Center, if not otherwise noted. Nuclear dynamics in embryos were visualized with a Histone2Av-RFP transgene (Kanesaki *et al.* 2011). Genomic transgenes generated in this study, *SHMT*[+]86F, *SHMT*[E130Q]86F, and *SHMT*[Ecoli]86F were marked with an associated *white*[+] and were generated by ϕC31 integrase-mediated site-specific insertions on the third chromosome at cytological position 86F8 (Bischof *et al.* 2007). Germline clones were induced by heat shock -induced expression of Flipase (2 × 1 hr, 37°, first, second instar larvae) and selected with *ovo*[D] with a matching Frt site (Frt[18E] or Frt[19A]). Mitotic clones in imaginal discs and follicle epithelium were induced by heat shock-induced expression of Flipase and negatively marked with nlsGFP. Embryos were collected from flies fed with yeast on apple juice plates kept at 25° for not longer than ∼2 wk. For the survival assay, 100 eggs were transferred from a staged collection to 55 mm petri dishes filled with standard fly food and incubated in a 25° incubator. The number of animals reaching the next developmental stage was scored. The genotypes were determined only in animals reaching the adult stage.

### Molecular cloning

Genomic constructs were cloned into the transformation vector pattB (Bischof *et al.* 2007). Details of the cloning procedures, sequences, and PCR protocols are available on request. Briefly, the *SHMT* locus was cloned as a *Bam*HI-*Bam*HI and a *Bam*HI-*Eco*RI from BAC PR98-75I5 into pattB. For cloning of variants, a *Pac*I site was introduced substituting the coding sequences including introns (pattBminusCDS). SHMT from *Escherichia coli* (*glyA*) from genomic *E. coli* DNA and a *SHMT* fragment comprising the region between the two alternative start codons were amplified by PCR and cloned into the *Pac*I site of pattBminusCDS. The E130Q mutation was introduced by site-directed mutagenesis. The SHMT expression plasmid was generated by inserting the PCR-amplified *SHMT* coding sequence lacking the N-terminal presequence as a *Nco*I-*Bgl*II fragment into pGEX-His6. The resulting construct (GST-SHMT-His6) has an N-terminal GST domain and a C-terminal 6 × histidine tail. For RNA *in situ* hybridization, *slam* and *kuk* RNA probes were generated with T7 RNA polymerase and antisense templates as previously described (Wenzl *et al.* 2010). For sequencing of the 5C region, genomic DNA was isolated from heterozygous adult flies. DNA of the region was amplified by PCR in pieces of a few kilobases and sequenced. The genomic DNA from another mutant derived from the same mutagenesis experiment (X287/FM7) served as a control to identify single nucleotide polymorphisms.

### NanoString genome-wide expression analysis

Single dechorionated embryos expressing Histone2Av-RFP were frozen 3 min after anaphase of the preceding mitosis in heptane on dry-ice. Three to five embryos of the same stage were pooled. “Late cycle 13” were *SHMT* embryos 25 min after anaphase of mitosis 12. This time corresponds to the approximate length of cycle 13 in wild-type embryos plus 3 min. For genome-wide analysis, embryos were collected according to the time after egg lay. Total RNA was extracted with Trizol (Invitrogen) and analyzed by Bio-analyzer (RNA 6000 Nano kit; Agilent). The yield was ∼50–150 ng total RNA per embryo. A set of selected transcripts was quantified by NanoString nCounter technology according to the protocol of the manufacturer. 50 ng of total RNA was subjected to analysis. Full original data sets for NanoString are available on request. For genome-wide analysis, RNA was subjected to next-generation sequencing (Illumina HiSeq2000) according to the manufacturer’s protocol, and 20–40 million reads per sample were sequenced with an annotation rate of > 90%. The highest number of reads reached > 10^5 for EF1α. Analysis was performed in biological triplicates. Genes with a low averaged number of reads (*N* < 50) in all four experimental conditions were not considered. The next-generation sequencing data have been deposited in NCBI’s Gene Expression Omnibus (Edgar *et al.* 2002) and are accessible through GEO Series accession number GSE97557 (https://www.ncbi.nlm.nih.gov/geo/query/acc.cgi?acc=GSE97557).

### SHMT antibody

GST-SHMT-His6 was expressed from the plasmid pGEX-SHMT-His6 in *E. coli* (BL21DE3). Following induction with 0.1 mM IPTG at 20° overnight in a 500 ml culture, the cleared supernatant from lysed cells was applied to a 1 ml HisTrap HP column. After washing the column, elution with imidazole-containing buffer, and desalting with a PD-10 column, ∼10 mg SHMT protein in 4 ml buffer were obtained. Rabbits and guinea pigs were immunized with the purified protein. Sera from both species gave comparable results in western blots and embryo staining.

### Western blots

SDS polyacrylamide gel electrophoresis and western blotting were performed as previously described (Wenzl *et al.* 2010). Briefly, for embryonic extracts, dechorionated and frozen embryos were boiled in 1 × Laemmli solution for 5 min. The cleared solution was stored at –20°. Extracts were subjected to SDS polyacrylamide gel electrophoresis and transferred to nitrocellulose membrane by semidry transfer. Following staining with Ponceau red and blocking with 5% bovine serum albumin in PBT [phosphate-buffered saline (PBS) with 0.1% Triton X-100], primary antibodies were added and incubated overnight at 4°. After washing (3 × rinsed, 4 × 10 min in PBT), the filter was incubated with secondary antibodies for 1.5 hr at room temperature. Primary antibodies were: rabbit/guinea pig-α-SHMT (this study). Western blots were developed with fluorescently-labeled secondary antibodies (800CW Donkey anti-guinea pig/rabbit IgG) and detected with a LICOR system. Sixteen-bit images were processed by Photoshop and FIJI/ImageJ.

### Gel filtration

Staged embryos (1.5–2.5 hr) were lysed in RIPA buffer [10 mM Tris/HCl [pH 7.5], 150 mM NaCl, 0.1% SDS, 1% Triton X-100, 1% deoxycholate, 5 mM EDTA, 2 mM PMSF, 1 × protease inhibitor cocktail (Roche), and 2 mM PMSF] with a Dounce homogenizer to a density of 10 embryos/μl. The cleared sample was applied to a Superdex 200HR 10/30 column preequilibrated with RIPA buffer. Proteins from collected fractions were dissolved in 6 × Laemmli sample buffer and analyzed by SDS polyacrylamide gel electrophoresis and western blot. The column was standardized by a set of marker proteins with known native molecular weight.

### Histology

Embryos were fixed in 4% formaldehyde in PBS for 20 min. Antibody staining and fluorescent RNA *in situ* hybridization was performed as previously described (Wenzl *et al.* 2010). In brief, after the posthybridization washes, embryos were incubated twice in PBT (PBS + 0.1% Tween-20) with 1% bovine serum albumin for 20 min each at room temperature, and then incubated for 2 hr with α-digoxigenin antibody coupled to peroxidase (Roche). Then, embryos were rinsed 3 × and washed for 4 × 15 min with PBT. The staining reaction was started by adding 200 µl of reaction solution (TSA-Cy3 diluted 1:200 in reaction buffer; PerkinElmer) to the embryos for 5–10 min. The reaction was terminated by adding 1 ml PBT. Embryos were washed once more with PBT. Protein staining with a specific antibody was performed after RNA staining.

S2 cells were fixed and stained as previously described (Wenzl *et al.* 2010). For cytoplasmic extraction, cells were washed in PBT, extracted with CSK buffer [10 mM Pipes/HCl (pH 6.8), 100 mM NaCl, 300 mM sucrose, 3 mM MgCl_2_, 1 mM DTT, 0, 2 mM PMSF, and 1 × protease inhibitor cocktail (Roche)] with 0.1% Triton X-100 and nuclease for 10 min on ice, washed with PBT, and fixed. The following antibodies were used. Primary antibodies: rabbit/guinea pig-α-SHMT (this study), rabbit/guinea pig-α-Slam (Wenzl *et al.* 2010), mouse-α-Dlg (4F3, Hybridoma center), rabbit-α-Frs (Grosshans *et al.* 2003), rabbit/guinea pig-α-Kuk (Brandt *et al.* 2006), rabbit-α-Dia (Grosshans *et al.* 2005), mouse-α-LaminDm0 (T47/1/1, Risau *et al.* 1981), mouse-α-γTubulin (GTU-88; Sigma), rabbit-α-CID (Jäger *et al.* 2005), rat-α-Twine (DiTalia *et al.* 2013), and GFP-booster (Chromotek). Secondary antibodies: Alexa-coupled goat-α-mouse/rat/rabbit/guinea pig antibodies (Invitrogen). DNA was stained by DAPI. Specimens were mounted in Aquapolymount (Polyscience).

### Clones

Mitotic clones were induced by 2 × 1 hr heat shocks at 37° in first and second instar larvae. Imaginal discs were prepared from third instar larvae in PBS, fixed in 4% formaldehyde for 40 min, and stained with GFP-booster. For clones in follicle epithelia, adult flies were heat shocked (2 × 1 hr at 37°). Ovaries were dissected on ice 3–5 d later, fixed in 4% formaldehyde/PBS for 20 min, and stained with SHMT antibody and GFP-booster.

### EdU labeling

Embryos (0.5–2.5 hr) were dechorionated with hypochlorite and air dried for 3–5 min. Following permeabilization with heptane for 3 min (as a monolayer in an iron mesh), the embryos were washed and incubated in Schneider’s medium containing 50 mM EdU at 25° for 15 min. After this labeling period, the embryos were fixed with 4% formaldehyde in PBS/heptane for 20 min. The fixed embryos were stored in methanol at –20°.

### Microscopy: live imaging

Fluorescent images of fixed and immunostained embryos were recorded with a confocal microscope (Zeiss LSM780, 25 × /NA0.8 and 40 × /NA1.2). For live imaging, embryos were dechorionated with hypochlorite for 90 sec, washed and lined up on a piece of apple juice agar, transferred to a coverslip coated with glue (“Tesa pack” tape dissolved in heptane), desiccated if necessary, and covered with halocarbon oil (Voltalef 10S). Time-lapse imaging of embryos was conducted on an inverted microscope with differential interference contrast optics (AxioCam camera) or with fluorescent optics and a spinning disc (Zeiss ObserverZ1, CSU-X1, AxioCam MRm, 25 × /NA0.5, 40 × /NA1.3, and 63 × /NA1.3). Images were processed with ImageJ/Fiji and Photoshop (Adobe).

### Data availability

All data, materials, and fly stocks described and used in this study are available on request. Accession numbers for available data in public databases: GEO accession number GSE97557.

### Essential and nonautonomous function of SHMT

**Figure 2:**
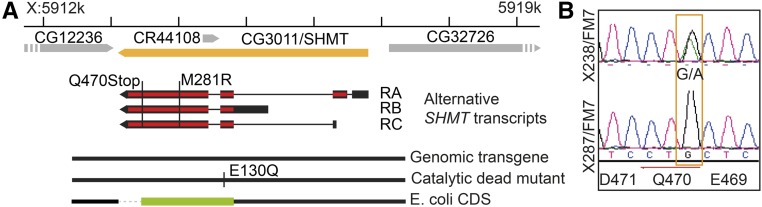
Lethal mutations in *SHMT*. (A) Genetic map of the *SHMT* locus with the annotation of three alternative transcripts encoding two protein isoforms according to FlyBase. The point mutations of the alleles *SHMT*[X238] (Q470stop) and *SHMT*[A] (M281R) are marked and genomic transgenes are indicated. The *SHMT* coding sequence from *E. coli* substituting the *Drosophila* coding sequence (CDS) is marked in green. (B) Traces from sequencing reactions of the *SHMT*[X238] allele in comparison to another mutation (X287) from the same mutagenesis screen.

We isolated a lethal point mutation, X238, in the *SHMT*/CG3011 gene (in the following designated as *SHMT*[X238]) from a collection of mutations with defects in early embryonic development (Figure 2A) (Vogt *et al.* 2006). Embryos and larvae derived from SHMT[X238] heterozygous parents developed normally until the pupal stage, when a fraction died as “black” pupae (∼20% of total) as compared to animals carrying a genomic rescue transgene (Supplemental Material, Figure S1A in File S1). No obvious defects were observed in the dying pupae (Table S4).

Embryos from females with germline clones of *SHMT*[X238] developed apparently normally until the blastoderm stage, but did not undergo cellularization and did not further develop. The complete failure of cellularization and formation of an epithelium is consistent with the observation that no structured cuticle material was formed during embryonic development (Figure S1B in File S1). As this is a fully penetrant phenotype, zygotic expression of *SHMT* in zygotically heterozygous female embryos derived from *SHMT* germline clones cannot rescue the early cell cycle and cellularization defects.

We mapped the lethality and germline clone phenotype on the X238 chromosome by meiotic recombination between *cv* and *sn*. In this manner, an associated male-sterile mutation was removed. Complementation with duplications and deficiencies narrowed down the position of the mutation to the 5C region including four genes (Figure S2 in File S1). Sequencing revealed a nonsense mutation (G→A) leading to a premature stop codon (Q470Stop, long protein; Q400Stop, short protein) in *SHMT* (Figure 2B). The mutation did not complement the lethality of an existing missense allele *SHMT*[M281R] (Haelterman *et al.* 2014). Furthermore, a 4883 bp genomic transgene with the wild-type allele complemented the lethality and germline clone phenotype of *SHMT*[X238] (Figure 2A and Table 1).

**Table 1:** Complementation of *SHMT*

		Males		
Transgene	Females	FM7	X0	Rescued
	*y pn SHMT*[*X238*] *sn* Frt[19A]/FM7 ♀♀ X ♂♂ *w*/Y; transgene{*w*+}			
WT rescue	234	90	0	117
*E. coli* SHMT	360	103	0	125
E130Q	113	42	1	0
	*w SHMT*[*X238*] *f* Frt[18E]hs-Flp/FM7 ♀♀X♂♂ *w*/Y; transgene{*w*+}			
WT rescue	373	68	0	146
E130Q	549	105	7	0
	*y SHMT*[*M281R*] Frt[19A]/FM7 ♀♀X♂♂ *w*/Y; transgene{*w*+}			
WT rescue	691	212	0	315
E130Q	712	191	0	280

WT, wild-type; SHMT, serine hydroxymethyl transferase.

*SHMT* is assumed to function as an enzyme that is central to C1 metabolism. Alternatively, SHMT protein may constitute a pseudoenzyme with a function in maintaining cellular structure. To test whether its enzymatic activity is required for gene function, we introduced a point mutation (E130Q) at a residue at the active center that is essential for catalytic activity. The E130Q point mutation does not severely affect the structure of the protein (Rao *et al.* 2000; Szebenyi *et al.* 2004). A transgene with this allele did not complement *SHMT*[X238], indicating that the enzymatic function is required. Interestingly, the *SHMT*[E130Q] transgene complemented the *SHMT*[M281R] allele. SHMT is assumed to act as a tetramer (Jagath *et al.* 1997; Bhatt *et al.* 2004). The E130Q and M281R mutations might thus affect different aspects of the enzymatic function.

These experiments do not rule out a structural function of SHMT in addition to an enzymatic function. To test the structural option, we varied the structure by inserting the *SHMT* gene from *E. coli* into a genomic transgene. The *E. coli SHMT* coding sequence was inserted in a manner in which two proteins, one with and one without the putative mitochondrial presequence, are produced. This transgene (*SHMT*[bact]) complemented the lethality and germline clone phenotype of *SHMT*[X238] (Figure 2A and Table 1). We conclude that *SHMT* may largely act via its enzymatic function.

**Figure 3:**
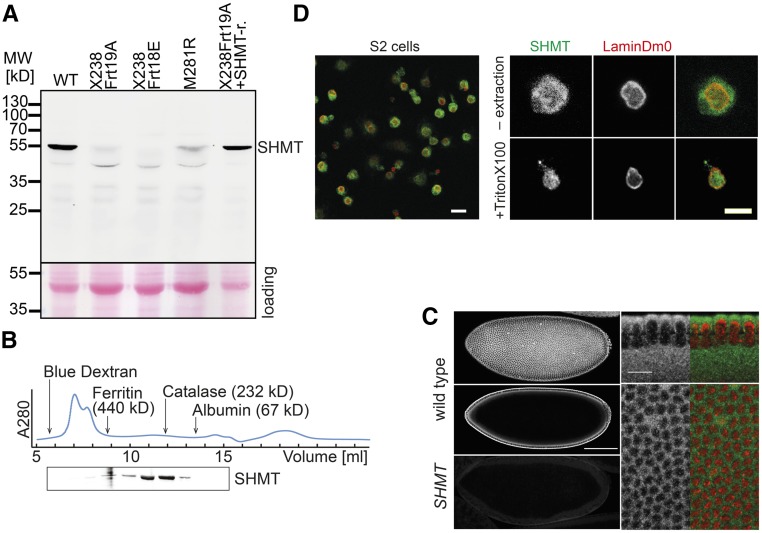
Serine hydroxymethyl transferase (SHMT) protein. (A) Western blot with embryonic extracts (0–3 hr) from wild-type (WT), *SHMT* alleles X238 (recombined with either Frt18E or Frt19A), M281R, and rescued embryos (X238 allele with a genomic rescue transgene). Loading control by Ponceau staining (red) following transfer. MW, molecular weight marker. (B) Gel filtration with embryonic extracts analyzed by western blotting of eluted fractions for SHMT protein. Elution peaks of molecular weight markers and the elution profile (A280) are indicated. (C) Confocal images of fixed wild-type and embryos from SHMT[X238] germline clones were stained with SHMT antibody (gray/green) and 4’,6-diamidino-2-phenylindole (DAPI, red). Bar, 100 and 10 µm. (D) Confocal images of fixed *Drosophila* S2 cells stained for SHMT (gray/green), LaminDm0 (gray/red). Extraction with Triton X-100 was performed prior to fixation. Bar, 10 and 5 µm.

According to transcript analysis of the gene and genome annotation, the *SHMT* gene is predicted to encode two proteins: a longer one of 470 amino acid residues with a putative N-terminal mitochondrial presequence, and a shorter one of 400 amino acid residues (Figure 2A). The predicted mitochondrial presequence is usually cleaved, which may lead to a mature mitochondrial protein with a size comparable to the predicted cytoplasmic isoform. We raised antibodies against a purified recombinant protein without the putative mitochondrial presequence. In immunoblots, antibodies from rabbit and guinea pig recognized a single protein band of ∼55 kDa in extracts from wild-type and SHMT[X238] embryos rescued with a *SHMT*[+] genomic transgene (Figure 3A). As we detected only one band in western blots, the predicted cytoplasmic and mitochondrial isoforms may have comparable sizes. In embryonic extracts from *SHMT*[X238] germline clones, the prominent 55 kDa band was absent or at least strongly reduced. These data indicate that the C-terminally truncated protein from the *SHMT*[X238] allele is unstable. With extracts of the *SHMT*[M281R] allele, a band with reduced intensity was detected. SHMT forms a dimer in *E. coli* and a tetramer in eukaryotes (Jagath *et al.* 1997; Bhatt *et al.* 2004). We assayed the native molecular weight of SHMT by gel filtration of cytoplasmic extracts. We detected SHMT in fractions corresponding to a molecular weight of globular proteins with ∼250 kDa (Figure 3B). These data indicate that SHMT largely constitutes a tetramer in *Drosophila*.

The antibody is specific in histological staining of fixed embryos. Consistent with the western blot, staining of fixed wild-type and *SHMT*[X238] embryos with SHMT antibodies showed a clear difference with staining almost absent in mutant embryos. In wild-type embryos, we detected uniform cytoplasmic staining during blastoderm stages (Figure 3C) and in later developmental stages. In cultured S2 cells, a uniform signal throughout the cells was detected (Figure 3D).

SHMT has been reported to associate with the nuclear envelope and DNA replication complexes (Anderson *et al.* 2012). In order to detect a minor nucleoplasmic fraction as compared to a prominent cytoplasmic SHMT pool, we extracted cultured S2 cells with Triton X-100. Triton X-100 extraction depletes the cytoplasm but leaves nuclei relatively intact. Staining of such extracted cells revealed a nucleoplasmic staining for SHMT together with the lamina protein LaminDm0 (Figure 3D). These data suggest that a subfraction of SHMT is present in the nucleus in *Drosophila* S2 cells.

### Nonautonomous function of SHMT

**Figure 4:**
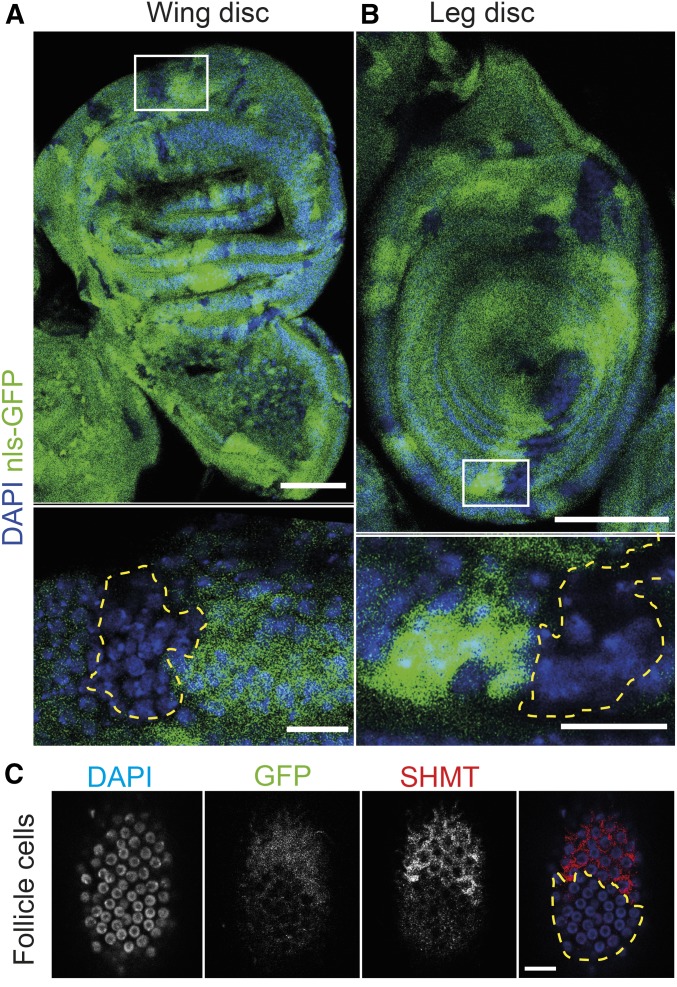
*SHMT* clones in epithelial tissues. Images of (A) wing and (B) leg imaginal discs with *SHMT*[X238] mutant clones were fixed and stained for DNA (blue) and GFP (green). Bar, 50 and 10 µm. (C) Image of follicle epithelium with *SHMT*[X238] clones fixed and stained for SHMT (gray/red), GFP (gray/green), and DAPI (blue). Absence of GFP or SHMT marks mutant clones (dashed line in yellow). Bar, 10 µm. DAPI, diamidino-2-phenylindole; GFP, green fluorescent protein; SHMT, serine hydroxymethyl transferase.

Given its enzymatic function in the mitochondria, cytoplasm, and potentially the nucleus inside the cell, SHMT may have an autonomous function. However, many metabolites are able to cross cell borders due to the presence of specific canals and transport proteins in the plasma membrane. To distinguish these options, we induced *SHMT* mutant clones as marked by the absence of GFP in leg and wing imaginal discs and the follicle epithelium in ovaries (Figure 4). In all three tissues, clones of *SHMT* mutant cells were frequently observed. This indicates that cells within a tissue can grow and pass through the cell cycle even in the absence, or at least strongly reduced amounts, of SHMT.

These observations are consistent with the availability of *SHMT* mutant embryos from germline clones. Here, *SHMT* homozygous oocyte and nurse cells grow in an environment of heterozygous follicle cells within a *SHMT* heterozygous female. Given the growth of egg chambers, it is unlikely that persistence of residual amounts of SHMT protein in the mutant nurse cells and oocyte fulfils the SHMT function. More likely is the uptake of SHMT-dependent metabolites from the follicle epithelium. The apparent nonautonomous function of *SHMT* may thus be explained by a transfer of SHMT-dependent metabolites across cell borders and uptake from extracellular fluid.

### SHMT is required for progression through nuclear division cycle 13 and cellularization

**Figure 5:**
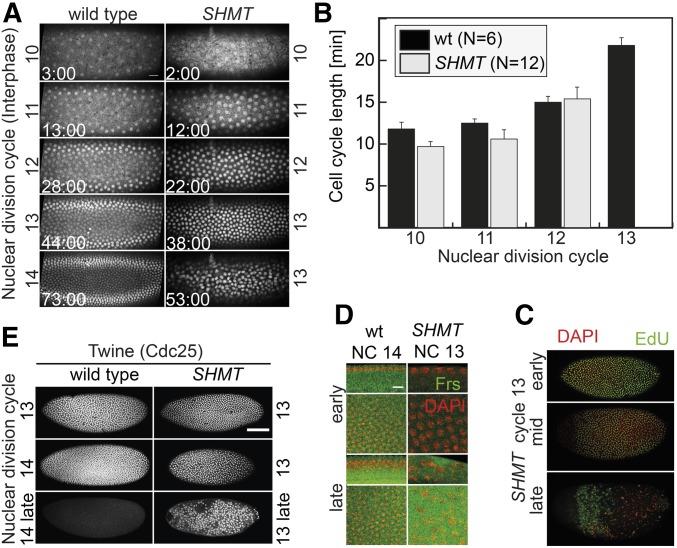
Cell cycle arrest in interphase 13 of *SHMT* mutants. Wild-type embryos and embryos from *SHMT*[X238] germline clones. (A) Fluorescent images from time-lapse recording of embryos expressing histone2Av-RFP. Time in minutes:seconds. Bar, 20 µm. (B) Corresponding quantification of cell cycle lengths. Error bar indicates SEM. Images of embryos fixed and stained for (C) Twine/Cdc25 or (D) Frs (green) and DAPI (red). Bar, 100 µm (C) or 10 µm (D). (E) DNA synthesis was marked by incorporation of EdU for 15 min. Fixed *SHMT* embryos (onset of cycle 13, early cycle 13, late stage) were stained for EdU (green) and DAPI (red). Bar, 100 µm. DAPI, diamidino-2-phenylindole; EdU, 5-ethynyl-2’-deoxyuridine; GFP, green fluorescent protein; NC, nuclear division cycle; RFP, red fluorescent protein; SHMT, serine hydroxymethyl transferase; wt, wild-type.

During early development, embryos pass through invariantly 13 rapid nuclear division cycles without cytokinesis giving rise to a syncytial embryo, in which all nuclei share a common cytoplasm. Following mitosis 13, a layer of epithelial cells form during cellularization. To describe cell cycle progression in wild-type and mutant embryos, we conducted time-lapse recordings of embryos expressing a RFP-tagged histone2Av to fluorescently label chromatin (Figure 5A). We did not detect differences during the first 12 cell cycles (Figure 5B). In contrast to wild-type embryos, which proceeded in a timely fashion through mitosis 13 and cellularized in interphase 14, the cell cycle in most mutant embryos arrested in interphase 13 (Figure 5B and Table 2).

**Table 2:** Penetrance of the cell cycle and cellularization phenotypes

	Arrest in Cell Cycle (in %)		
Genotype	13	14	*N*
Wild-type	0	100	7
*SHMT*[X238] Frt[19A]	91.7	8.3	24
*SHMT*[M281R] Frt[19A]	76.2	23.8	21
	Cellularization (%)		
Genotype	No/partial	Complete	*N*
Wild-type	0	100	7
*SHMT*[X238] Frt[19A]	95.8	4.2	24
*SHMT*[M281R] Frt[19A]	70	25	20

In addition to the behavior of chromatin as a marker for cell cycle progression, we stained for centrosomes, which duplicate early in interphase, and for centromeres (Figure S3 in File S1). We found two centrosomes for most of the nuclei (Figure S3A in File S1). However, in later stages of mutant embryos, the association of nuclei and centrosomes was broken due to widespread nuclear fallout. The number of centromeres seemed to be normal in *SHMT* mutants within the resolution of our assay (Figure S3B in File S1). Immediately after entering interphase, DNA replication starts during the fast nuclear division cycles, which lack gap phases. In *SHMT* mutants, we detected uniform DNA synthesis in all nuclei early in cycle 13 (Figure 5C), similar to wild-type embryos. Later, the staining pattern for DNA synthesis became irregular, with single nuclei lacking staining. Late stage embryos with already severe morphological defects still showed staining for DNA synthesis in some areas of the embryos, indicating a persisting S phase.

In wild-type embryos, the cell cycle pauses in interphase 14 due to degradation of Twine/Cdc25 protein and expression of zygotic inhibitor Frühstart (Frs) (Farrell and O’Farrell 2013; DiTalia *et al.* 2013; Grosshans *et al.* 2003; Gawliński *et al.* 2007). Through immunohistology, we found that both mechanisms were not precociously activated in *SHMT* mutant embryos (Figure 5, D and E). Frs is expressed at the onset of interphase 14 and inhibits Cdk by binding to Cyclin (Grosshans *et al.* 2003; Gawliński *et al.* 2007). In *SHMT* mutant embryos, Frs protein was detected only in very late embryos, as staged by the severe morphological defects (Figure 5D). Twine staining was detected in *SHMT* mutant embryos even at late stages, which showed severe morphological defects, whereas Twine was already strongly reduced in wild-type embryos at midstage cellularization (Figure 5E). Our data indicate that *SHMT* mutant embryos specifically arrest in interphase 13 and miss the last nuclear division in mitosis 13. The cell cycle arrest is not due to precocious degradation of Twine/Cdc25 or expression of Frs.

**Figure 6:**
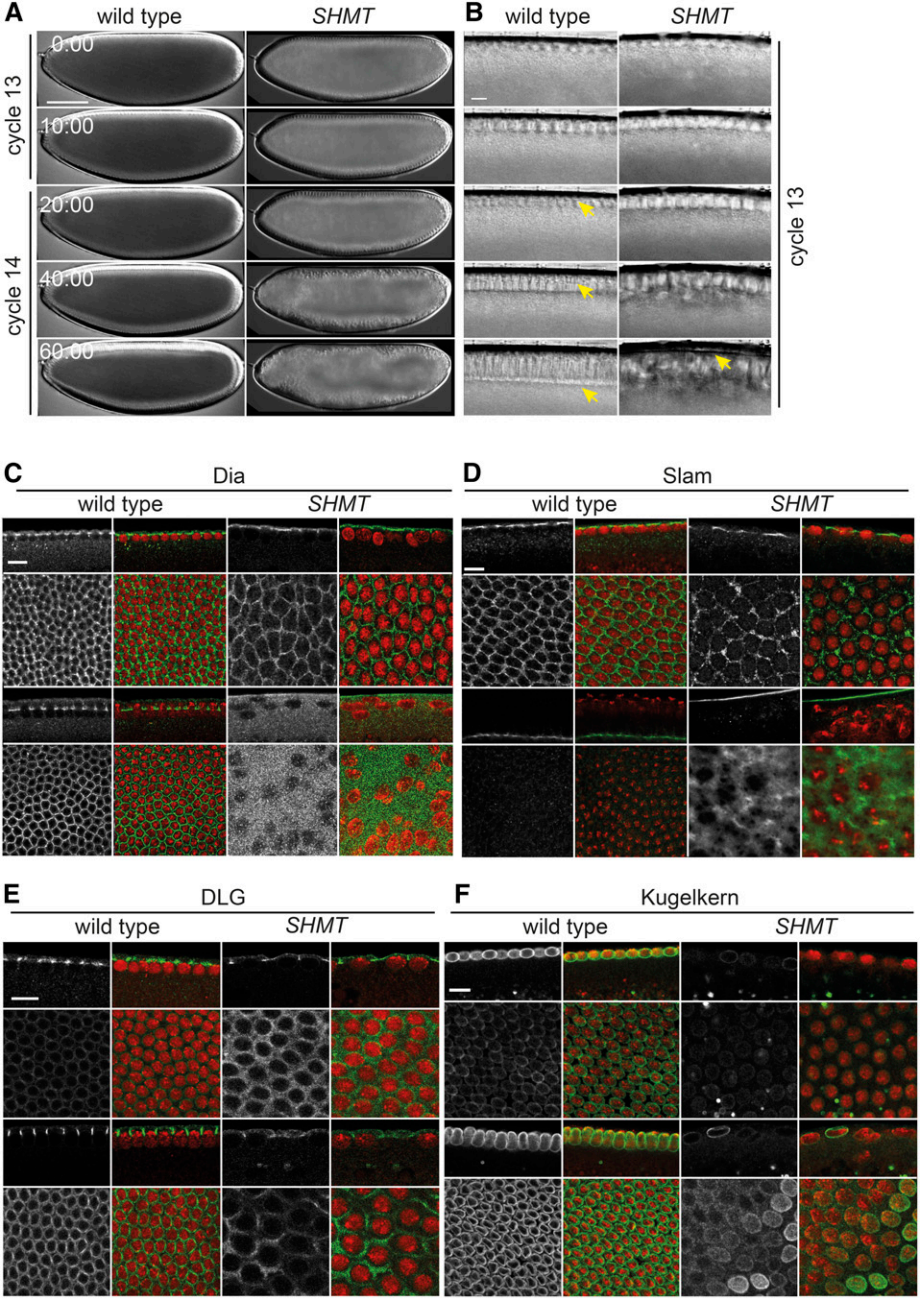
Cellularization phenotype of *SHMT* mutants. Wild-type embryos and embryos from *SHMT*[X238] mutant germline clones. (A and B) Live images by visible light microscopy (differential interference contrast) at (A) low and (B) high magnification. Time in minutes:seconds. Bar, 100 µm (A) or 10 µm (B). Arrow in yellow points to cellularization furrows. (C–F) Confocal images of embryos in early and advanced stage of cycle 14 or 13, fixed and stained as indicated. DNA (red) Bar, 10 µm. (C) Dia, (D) Slam, (E) Dlg, and (F) Kuk (gray/green).

Concomitantly with the cell cycle pause in interphase 14, cellularization proceeds. In this process, the plasma membrane invaginates between adjacent nuclei to enclose them in individual cells. At the same time, the spherical nuclei transform into an extended shape (Brandt *et al.* 2006). In the case of a premature cell cycle pause in cycle 13, cellularization may proceed (Sung *et al.* 2013; Grosshans *et al.* 2003). The invaginating membrane is visible as a progressively extending furrow between the nuclei in light microscopy (Figure 6A). In *SHMT* mutants, neither furrow nor nuclear elongation were visible (Figure 6B). The nuclei acquired an irregular shape and lost their regular cortical alignment.

The observations of visible morphology were confirmed by protein markers, Dia and Slam, which both associate with the furrow tip throughout cellularization in wild-type (Grosshans *et al.* 2005; Wenzl *et al.* 2010). In contrast, Dia and Slam were uniformly distributed or cortically localized in *SHMT* mutants (Figure 6, C and D). Dlg, a marker for the lateral membrane, localized at the cortex and did not mark any furrow-like structure in *SHMT* mutants (Figure 6E). The protein Kugelkern (Kuk) localizes at the inner nuclear membrane and is required for nuclear elongation (Brandt *et al.* 2006). Expression of Kuk is initially low but increases at the onset of cellularization. Kuk staining was weak in early cycle 13 as expected. Later, in embryos with clear morphological defects, an upregulation was detected in only some nuclei, as shown by the strong Kuk staining (Figure 6F). Taken together, our immunohistological analysis shows that *SHMT* mutant embryos do not pass through cellularization.

### Gene expression in SHMT mutants

**Figure 7:**
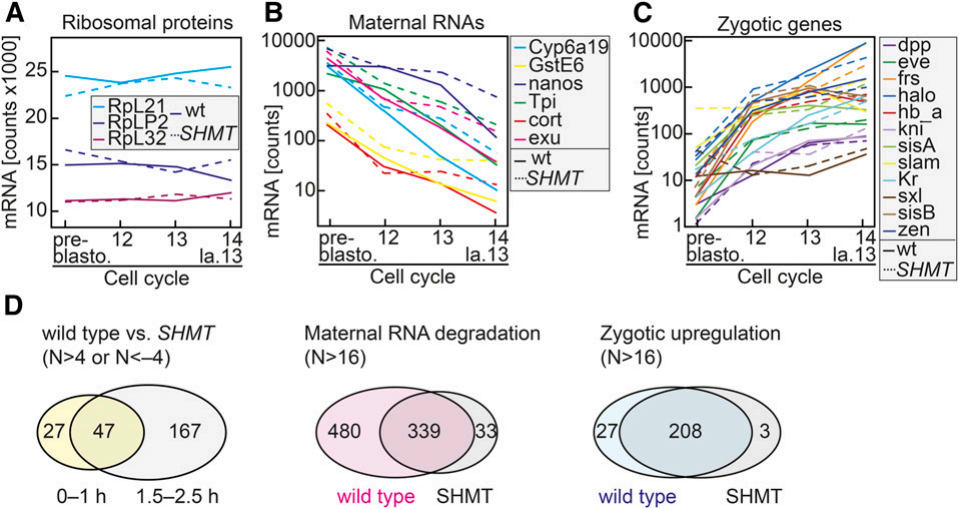
Gene expression in *SHMT* mutants. (A–C) Level of representative transcripts assayed by NanoString analysis with RNA samples from wild-type and *SHMT*[X238] embryos manually staged according to the nuclear division cycle. Late 13 in *SHMT* mutants corresponds to time of cycle 14 collection in wild-type. (A) Ribosomal proteins, (B) RNAs subject to maternal degradation (logarithmic scale), and (C) early zygotic genes (logarithmic scale). (D) Genome-wide analysis of differential transcript levels between wild-type and *SHMT*[X238] mutant embryos at 0–1 hr or 1.5–2.5 hr after egg lay and between preblastoderm (0–1 hr) and blastoderm (1.5–2.5 hr) stages. Selection of genes as indicated. FDR, false discovery rate; SHMT, serine hydroxymethyl transferase; wt, wild-type.

Cellularization depends on the onset of zygotic gene expression. Absence of zygotic gene expression prevents cellularization, whereas its precocious onset leads to cellularization in cycle 13 (Sung *et al.* 2013). We employed NanoString nCounter analysis for a selected set of genes with representative expression profiles to establish profiles of gene expression in precise temporal resolution. RNA samples were prepared from 3 to 5 embryos per sample, manually selected according to the nuclear division cycle. We detected very related expression profiles for wild-type and *SHMT* mutants for constitutively expressed genes (Figure 7A), RNAs subject to maternal degradation (Figure 7B), and zygotically expressed genes (Figure 7C). These measurements were consistent with the immunostainings for zygotic proteins (Slam, Kuk, and Frs; Figure 5 and Figure 6) and indicate that the gene expression program, including activation of zygotic transcription and degradation of maternal RNAs, is largely functional in *SHMT* mutants.

To validate this conclusion, we compared genome-wide changes in gene expression between wild-type and *SHMT* mutant embryos at the preblastoderm (0–1 hr) and blastoderm stages (1.5–2.5 hr) (Figure 7D). In 0–1 hr embryos (nuclear cycles 1–9), we detected significant changes between wild-type and *SHMT* embryos of more than fourfold in only 74 genes (Figure 7D and Table S1 in File S1). These genes did not fall into specific functional groups as judged by gene ontology annotations. The set includes genes with moderate expression in SHMT mutants but no or low expression in wild-type embryos, *e.g.*, the glutathione transferase GSTD3 and vice versa. The higher expression of *pn* in wild-type as compared to *SHMT* mutants served as a positive control, as the *SHMT* chromosome was marked with *pn*. Consistent with the nonautonomous function of *SHMT*, these data indicate that eggs from *SHMT* germline clones are almost like wild-type with respect to maternal RNAs. In contrast, > 214 genes had fourfold different expression levels in 1.5–2.5 hr embryos (Figure 7D and Table S1 in File S1). On the one hand, this set contains many transcripts that are normally degraded but were more stable in *SHMT* mutants. On the other, some genes were not expressed in *SHMT* mutants but moderately in wild-type embryos, similar to the positive control *pn*. The differences were not due to generally impaired transcription in *SHMT* mutants, since we detected strong changes in both directions, *i.e.*, up- and downregulation.

The onset of zygotic gene expression and the degradation of maternal RNAs are two important changes in transcript levels during the blastoderm stage. The set of the 819 transcripts subject to maternal degradation in wild-type embryos included the vast majority of the corresponding set (339 out of 372) in *SHMT* embryos (Figure 7D and Table S2 in File S1). The majority of the genes in the sets specific for either wild-type or *SHMT* mutants were also subject to maternal degradation in the other genotype but to a lesser extent, below our arbitrary threshold of 16 ×. The GstD3 gene falls out of this categorization, as it is not expressed in wild-type embryos but is subject to maternal degradation in *SHMT* mutants.

Out of the 235 zygotic genes with an at least 16-fold increase during the blastoderm stage in wild-type embryos, 208 genes showed also an at least 16 × increased expression in *SHMT* mutants (Figure 7D and Table S3 in File S1), indicating that activation of the zygotic genome is largely functional in *SHMT* mutants. The three genes with specific zygotic induction in *SHMT* mutants were also induced in wild-type embryos but with a factor below our threshold. The 27 genes with specific zygotic induction in wild-type embryos were also induced in mutants, but to a lesser extent.

*SHMT* embryos are characterized by impaired cellularization and precocious cell cycle pause. This might be due to failed expression of genes such as *slam* or Kinesin-1 (Acharya *et al.* 2014; Winkler *et al.* 2015), which are required for cellularization, or genes involved in the remodeling of the cell cycle during MBT, such as *twine*, *frs*, or *trbl*. In the list of zygotic genes expressed in wild-type embryos but not in *SHMT* mutants (Table S3 in File S1), we did not find genes with an assigned cellularization function. Correspondingly, no such candidate genes were found in the list of maternal genes (Table S2 in File S1). These data indicate that the observed cell cycle arrest phenotype and absent cellularization is not a simple consequence of failed expression of cellularization or a cell cycle gene, or impaired induction of zygotic gene expression, for example. Taken together, our data from both methods, NanoString with selected genes and genome-wide RNAseq, indicate that activation of zygotic gene expression and control of maternal RNA degradation do not largely depend on *SHMT*.

### Stress response in SHMT mutants

**Figure 8:**
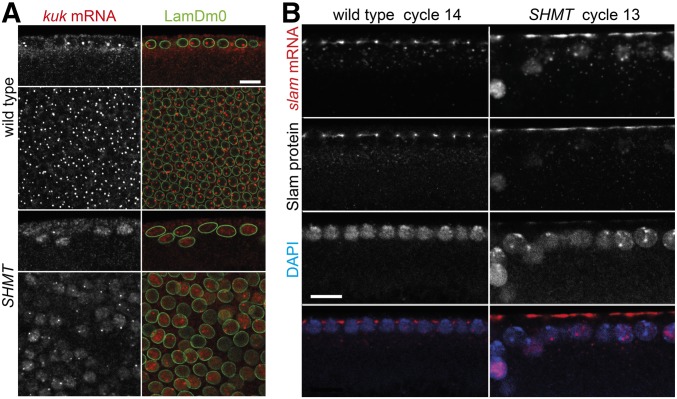
Nuclear retention of RNAs in *SHMT* mutants. Wild-type embryos and embryos from *SHMT*[X238] mutant germline clones fixed and stained for (A) *kuk* mRNA (gray/red) and LaminDm0 (green) or (B) *slam* mRNA (gray/red), Slam protein (gray), and diamidino-2-phenylindole (DAPI, gray/blue). Note that primary transcription sites in the nuclei are likely to be detected and *slam* mRNA colocalizes with Slam protein. Bar, 10 µm.

The impaired nuclear export of a set of mRNAs is a stress response in early embryos. Exposure to UV light elicits a Chk2-dependent nuclear retention of *kuk* mRNA, for example (Iampietro *et al.* 2014). We stained *SHMT* mutant embryos for *kuk* mRNA by fluorescent *in situ* hybridization (Figure 8A). As is typical for early zygotic genes, one or two nuclear foci were detected that probably represent the sites of transcription. Otherwise, *kuk* mRNA was detected in the cytoplasm. In contrast to wild-type embryos, clear nucleoplasmic staining was detected in some but not all nuclei in *SHMT* mutants, similar to what has been reported for embryos after UV exposure. In addition to *kuk* mRNA, we found that *slam* mRNA was retained in the nucleoplasm in *SHMT* mutants (Figure 8B). *slam* mRNA normally colocalizes with Slam protein at the tip of the invaginating furrow (Wenzl *et al.* 2010). In *SHMT* mutants, a nucleoplasmic signal was detected in some but not all nuclei in addition to the cortical signal. These data suggest that a stress response was elicited in *SHMT* mutants.

## Discussion

In genetic terms, *SHMT* serves a nonautonomous function. Clones of mutant cells proliferate when surrounded by wild-type cells, as investigated in leg and wing imaginal discs as well as the follicle epithelium. Similarly, mutant eggs grow in the environment of wild-type cells in the ovary. There are no indications that the SHMT protein itself is secreted from the cells. However, the metabolites that are dependent on SHMT are likely to cross the cell borders. Nonautonomy of essential metabolic enzymes has been reported previously, *e.g.*, the metabolite PAPS (Zhu *et al.* 2007).

SHMT is compartmentalized within cells. SHMT is present in mitochondria and the cytoplasm. This distinction is reflected by the presence of two separate genes in humans and mice (Anderson and Stover 2009; Anderson *et al.* 2011). *SHMT1* encodes the cytoplasmic isoform, *SHMT2*, the mitochondrial isoform. In *Drosophila*, a single gene encodes both isoforms. In addition to this mitochondrial–cytoplasmic compartmentalization, a third pool of SHMT may specifically act within the nucleus. Previous reports indicate that nuclear SHMT may be associated with the nuclear lamina and DNA replication complexes (Anderson *et al.* 2012). The nuclear pool of SHMT may be involved in local synthesis of thymine deoxynucleotides, which would be directly funneled into DNA synthesis. We observed nuclear staining of SHMT consistent with previous reports. Having a tractable genetic system with mutant and genomic rescue transgenes available, the relevance of the nuclear pool and lamina association of SHMT can now be investigated.

The developmental defect in embryos from *SHMT* germline clones appears to be very specific. Using several markers for chromatin, cell cycle, and cellular organization, we did not observe deviations from wild-type embryos prior to nuclear division cycle 13. According to our analysis, mitosis 12 was normal, including chromosome segregation, decondensation, centrosome duplication, and nuclear envelope reformation. This temporal specificity indicates that a single specific process becomes delayed or impaired in *SHMT* mutant embryos. As purines and dTTP are among the potential metabolites, DNA replication may be affected. In *SHMT* mutant embryos in interphase 13, incorporation of EdU was detected even in late stages, suggesting that DNA replication persists and is possibly delayed. A complete inhibition of DNA replication would cause a different phenotype. Inhibition of replication by aphidicolin does not arrest the centrosome cycle, for example (Raff and Glover 1988).

Impaired zygotic gene expression would lead to a different phenotype, *i.e.*, an additional nuclear division cycle (Foe *et al.* 1993; Sung *et al.* 2013). The changes in both directions also show that the phenotype is not a simple consequence of impaired maternal degradation. The conclusion that neither onset of zygotic transcription nor degradation of maternal RNAs are specifically affected is consistent with our analysis of representatives for early zygotic genes and RNAs subjected to maternal degradation by Nanostring analysis employing precisely staged embryos. A difficulty with the whole-genome analysis by RNAseq was staging of embryos. As larger amounts of material were required for the RNAseq method than for NanoString analysis, collection of embryos was conducted according to the time after egg lay. Thus, the 1 hr period (1.5–2.5 hr) that we used for collection included mutant embryos in late nuclear division cycles without morphological defects and embryos that already had severe morphological defects.

The blastoderm phenotype of *SHMT* mutants is reminiscent of embryos, in which the synthesis of deoxynucleotides is suppressed by hydroxyurea, an inhibitor of the nucleotide diphosphate reductase (Broadie and Bate 1991). Embryos treated with hydroxyurea develop without apparent defects until nuclear division cycle 12 (Blythe and Wieschaus 2015b, our own observations). Similarly, *Xenopus* embryos, treated with hydroxyurea, develop rather normal until MBT and arrest their cell cycle after cleavage division 12 (Vastag *et al.* 2011). Given that hydroxyurea-treated and *SHMT* embryos share similarities in their phenotypes, purine or pyrimidine deoxynucleotides dGTP, dATP, dCTP, or dTTP may become limiting in *SHMT* mutant embryos in interphase 13. One or more dNTP may be present in insufficient amounts in interphase 13 in *SHMT* mutants, which may lead to a delay in DNA replication, induce DNA replication stress, and cause checkpoint activation. Alternatively, SAM and a SAM-dependent methylation reaction may be rate-limiting and cause or contribute to the observed phenotypes.

Whatever the rate-limiting metabolite is in *SHMT* mutants, the mutant phenotype reveals that SHMT-dependent metabolites are provided by the egg in amounts sufficient for normal early development until the last nuclear cycle, which roughly coincides with the MBT. At this stage, the zygote has to generate these SHMT-dependent metabolites on its own. Defining the relation of maternally derived and zygotically synthesized metabolites, and thus the transition from maternal to zygotic metabolites, will require a detailed analysis of metabolite content and metabolic fluxes of precisely staged embryos. Although the cell cycle arrest in hydroxyurea-treated and *SHMT* embryos coincides with MBT, any functional role of deoxynucleotide or SHMT-dependent metabolite levels in timing of MBT would be purely speculative. In a developmental metabolite analysis in *Xenopus* embryos, Vastag *et al.* (2011) observed varied dynamic changes of deoxynucleotide levels prior to MBT and discussed conceivable roles of metabolite levels in MBT. The *SHMT* mutant embryos presented in this study may serve to identify metabolites that become rate-limiting in *SHMT* embryos and help to address the question of whether specific metabolites are involved in controlling the timing of MBT, as speculated by Vastag *et al.* (2011). In studying the mechanism of MBT, it may be worthwhile to consider changes in metabolites and metabolism beside the well-established features, such as zygotic genome activation, chromosomal reorganization, morphological changes, and cell cycle switch.

## Supplementary Material

Supplemental material is available online at www.g3journal.org/lookup/suppl/doi:10.1534/g3.117.043133/-/DC1.
