# Supplementary material for: Essential Function of the Serine Hydroxymethyl Transferase (SHMT) Gene During Rapid Syncytial Cell Cycles in Drosophila
Source: G3 (Bethesda). 2017 May 17;7(7):2305–14. doi: 10.1534/g3.117.043133 (PMC5499137; doi:10.1534/g3.117.043133)
Supplement: Supplementary file 1 [file 2305FileS1.pdf]

Supplemental data and material to:

F. Winkler, M. Kriebel, M. Clever, S. Gröning, J. Großhans. Essential function of the serine hydroxymethyl transferase (SHMT) gene during rapid syncytial cell cycles in Drosophila. Published in G3 (2017).

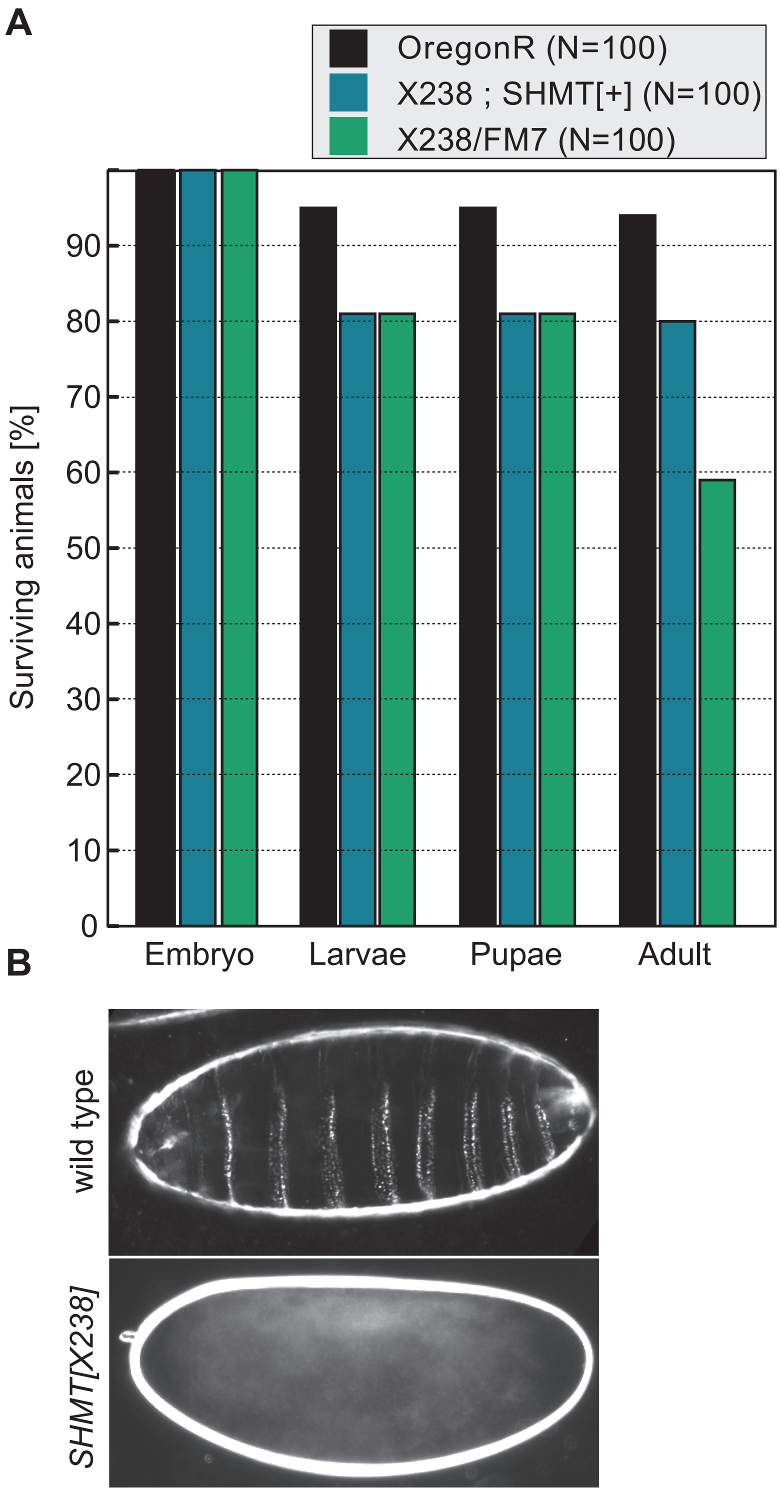

Supplemental data Figure S1: Mapping of the *SHMT*[X238] mutation. (A) The lethal phase of the *SHMT*[X238] mutation was determined by scoring the number of surviving animals as embryos, larvae, pupae or adult flies with indicated genotype. The experiment was started with each 100 eggs. Indicated is the number of animals from the previous stage. Note that the embryos in the *SHMT*[X238]/FM7 experiment were derived from heterozygous flies leading to three zygotic genotypes in a 1:2:1 were present. No *SHMT*[X238]/Y within the animals reaching adult stage. **(B)** Larval cuticle of wild type and embryo from *SHMT*[X238] germline clones. The cuticle phenotype was uniform in all embryos, and no zygotic rescue was observed in female embryos.

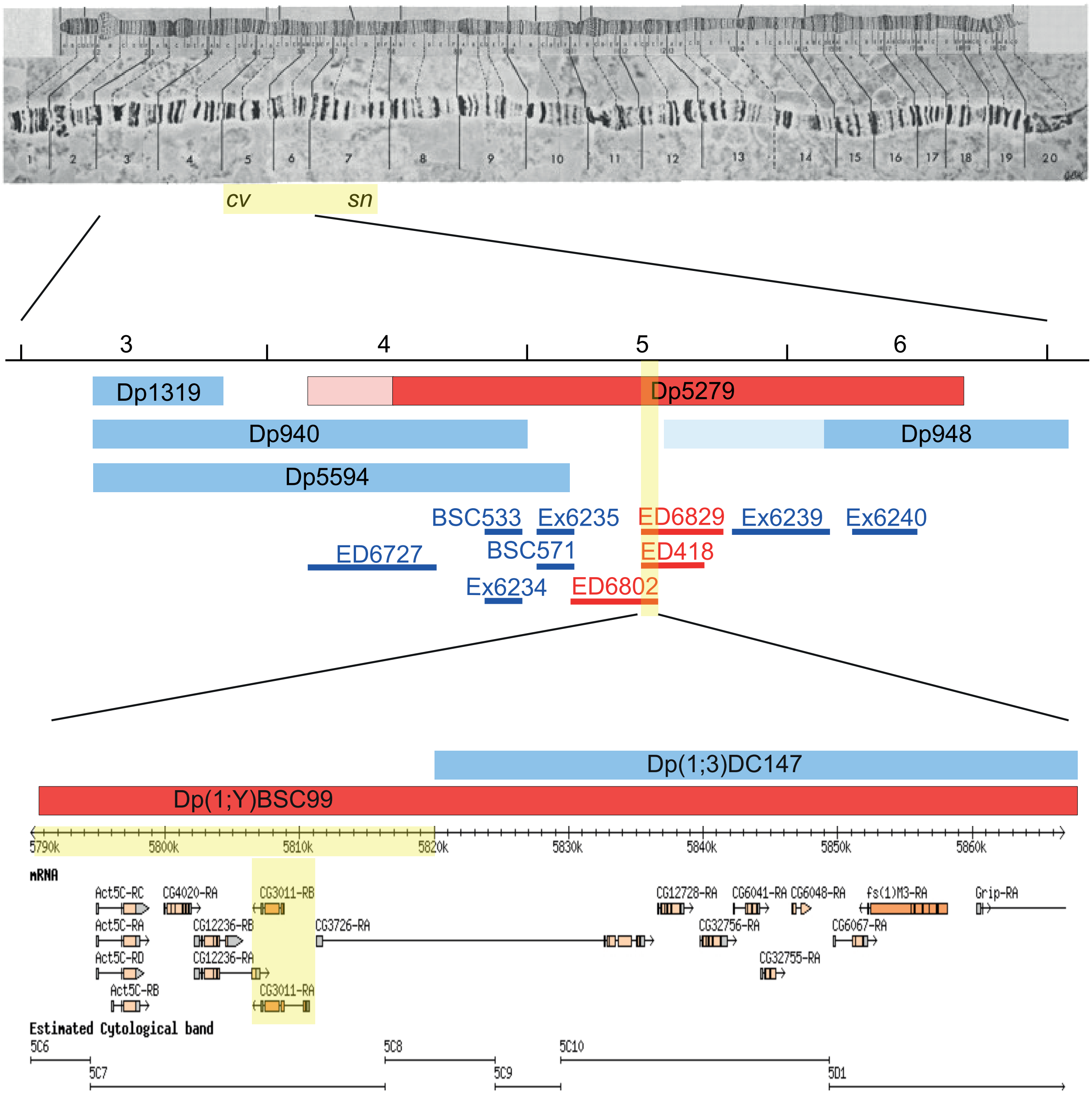

Supplemental data Figure S2: Mapping of the *SHMT*[X238] mutation. The lethality and germline clone phenotype of *SHMT*[X238] was mapped by meiotic recombination and complementation with duplications and deficiencies. Complementing duplications and non-complementing deficiencies are marked in red.

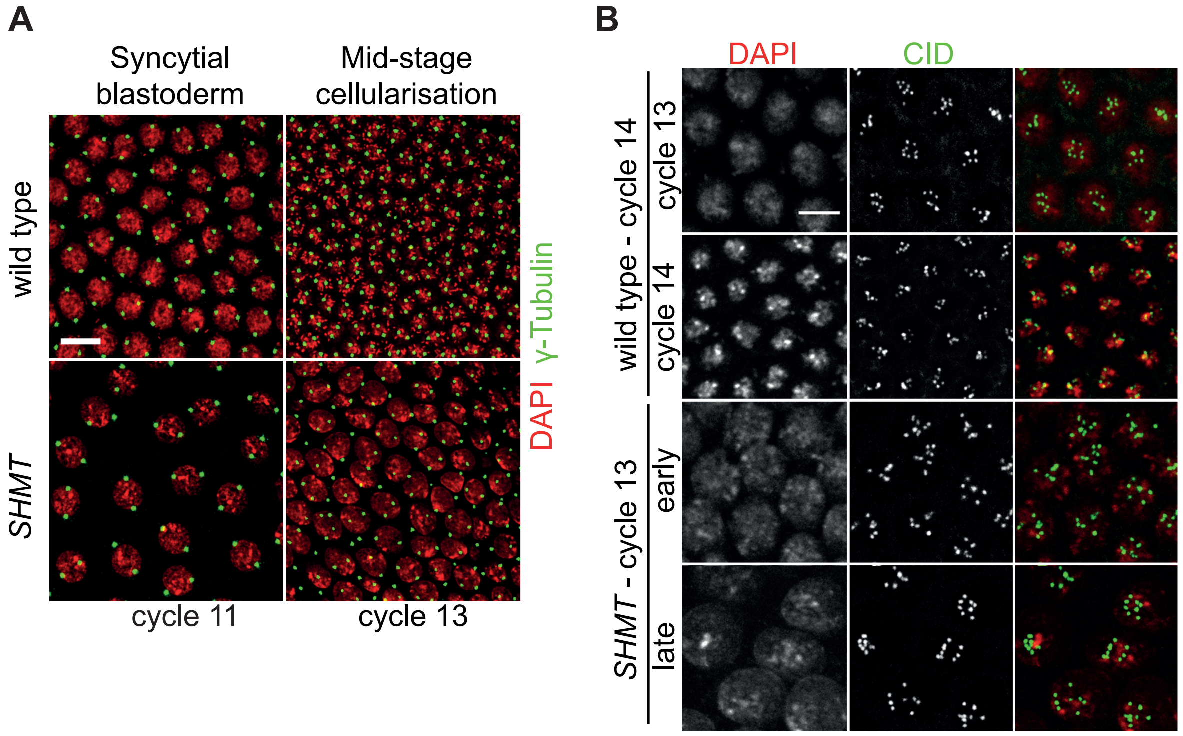

Supplemental data Figure S3: Cell cycle markers in *SHMT* mutants. Wild type embryos and embryos from *SHMT*[X238] mutant germline clones fixed and stained for **(A)** centrosomal marker γ-tubulin (green) and DAPI (red), **(B)** centromereal marker CID (grey/green), DAPI (grey/red). Scale bar 10 μm.

Supplemental data Table S1: Differential gene expression in wild type embryos and embryos from *SHMT*[X238] germline clones in preblastoderm stage (0–1 h) and in blastoderm stage (1.5–2.5 h). Average of three biological replicates.

Table S1: Difference in gene expression between wild type and SHMT mutants

Genes selected by difference during preblastoderm stage

Gene Id	Name	Difference WT–SHMT (log2) 0–1 h	Difference WT–SHMT (log2) 1.5–2.5 h	Gene Id	Name	Difference WT–SHMT (log2) 0–1 h	Difference WT–SHMT (log2) 1.5–2.5 h
FBgn0034788	CG13532	–8.59	–7.73	FBgn0003312	sad	2.55	1.02
FBgn0010039	GstD3	–7.94	–2.91	FBgn0000071	Ama	2.59	1.82
FBgn0038412	Zip89B	–7.93	–7.78	FBgn0002543	lea	2.65	2.83
FBgn0263219	Dscam4	–7.82	–6.34	FBgn0040045	CR12460	2.74	2.79
FBgn0035287	CG13937	–7.77	–6.57	FBgn0000576	ems	2.81	2.09
FBgn0033395	Cyp4p2	–7.14	–5.91	FBgn0004102	oc	2.91	1.5
FBgn0052581	CG32581	–6.9	–7.45	FBgn0003463	sog	2.97	1.81
FBgn0036652	CG13032	–6.39	–7.43	FBgn0261648	salm	3.01	3.13
FBgn0001311	kkv	–6.11	–0.52	FBgn0037213	CG12581	3.05	1.07
FBgn0038419	CG14879	–5.9	–7.15	FBgn0050163	Cpr60D	3.13	1.46
FBgn0262719	CG43163	–5.02	2.58	FBgn0011706	rpr	3.16	0.35
FBgn0052017	CG32017	–4.41	–4.15	FBgn0005558	ey	3.17	3.34
FBgn0262104	CG42857	–4.02	–6.36	FBgn0000606	eve	3.53	2.81
FBgn0038395	CG10407	–3.82	–2.78	FBgn0042627	v(2)k05816	3.58	3.18
FBgn0053319	CR33319	–3.81	–3.55	FBgn0259171	Pde9	3.69	3.65
FBgn0022359	Sodh-2	–3.66	–4.03	FBgn0003116	pn	3.72	5.05
FBgn0262636	Lin29	–3.55	–1.27	FBgn0032235	CG5096	4.01	1.66
FBgn0035563	CG13716	–3.4	–2.17	FBgn0040373	CG3038	7.64	7.46
FBgn0029856	CG11700	–3.15	–2.63	FBgn0085382	CG34353	7.87	7.15
FBgn0035770	pst	–3.11	–3.53	FBgn0263416		9.1	7.91

Genes selected by difference during blastoderm stage (1.5–2h)

Gene Id	Name	Difference WT–SHMT (log2) 0–1 h	Difference WT–SHMT (log2) 1.5–2.5 h	Gene Id	Name	Difference WT–SHMT (log2) 0–1 h	Difference WT–SHMT (log2) 1.5–2.5 h
FBgn0034788	CG13532	–8.59	–7.73	FBgn0042627	v(2)k05816	3.58	3.18
FBgn0052581	CG32581	–6.9	–7.45	FBgn0029907	Atx-1	0.57	3.21
FBgn0036652	CG13032	–6.39	–7.43	FBgn0037513	pyd3	2.27	3.28
FBgn0038419	CG14879	–5.9	–7.15	FBgn0005558	ey	3.17	3.34
FBgn0035287	CG13937	–7.77	–6.57	FBgn0260861	Trs23	0.3	3.46
FBgn0030938	CG15047	–0.62	–6.5	FBgn0259171	Pde9	3.69	3.65
FBgn0262104	CG42857	–4.02	–6.36	FBgn0034606	ASPP	1.33	3.69
FBgn0263219	Dscam4	–7.82	–6.34	FBgn0031313	CG5080	1.68	3.69
FBgn0033395	Cyp4p2	–7.14	–5.91	FBgn0010473	tutl	0.68	3.77
FBgn0037518	CG2641	–2.51	–5.3	FBgn0034756	Cyp6d2	0.72	3.78
FBgn0029706	CG3626	–0.26	–4.23	FBgn0002945	nkd	1.33	4
FBgn0052017	CG32017	–4.41	–4.15	FBgn0085412	CG34383	2.05	4.13
FBgn0030964	Pvf1	0.37	–4.08	FBgn0030816	CG16700	1.49	4.16
FBgn0263589	CR43614	–0.25	–4.05	FBgn0003002	opa	0.89	4.28
FBgn0022359	Sodh-2	–3.66	–4.03	FBgn0003116	pn	3.72	5.05
FBgn0031321	Tgt	0.28	–3.87	FBgn0035246	CG13928	7.26	5.16
FBgn0010097	gammaTub	–0.13	–3.78	FBgn0033108	CG15236	1.57	6.44
FBgn0032031	CG13390	0.77	–3.7	FBgn0085382	CG34353	7.87	7.15
FBgn0053319	CR33319	–3.81	–3.55	FBgn0040373	CG3038	7.64	7.46
FBgn0035770	pst	–3.11	–3.53	FBgn0263416		9.1	7.91

Supplemental data Table S2: Maternal transcripts downregulated between preblastoderm (0–1 h) and blastoderm stage (1.5–2.5 h) in wild type embryos and embryos from *SHMT*[X238] embryos. Average of three biological replicates.

Table S2: Genes with differential maternal degradation

Genes with strong maternal degradation (N>4) in either wild type or SHMT mutants

		wild type		SHMT		Difference
Gene Id	Name	log2	p value	log2	p value	WT–SHMT
FBgn0010039	GstD3	–0.15	9.1E–01	4.87	2.2E–73	–5.02
FBgn0011674	insc	1.42	2.1E–06	4.16	6.9E–19	–2.74
FBgn0030317	CG1561	2.22	7.2E–22	4.08	3.1E–29	–1.87
FBgn0026084	cib	2.61	5.8E–119	4.22	2.5E–214	–1.61
FBgn0015808	ScpX	2.66	1.7E–31	4.34	1.7E–42	–1.68
FBgn0001965	Sos	2.72	1.1E–15	4.28	6.7E–41	–1.55
FBgn0038872	Nelf-A	2.73	4.0E–21	4.36	3.2E–90	–1.63
FBgn0030968	CG7322	2.86	1.9E–14	4.89	8.9E–27	–2.03
FBgn0016754	sba	3.07	1.2E–33	4.74	3.2E–55	–1.67
FBgn0034853	Ice1	3.08	8.0E–22	4.04	7.0E–58	–0.96
FBgn0052529	Hers	3.14	2.4E–38	4.01	1.6E–41	–0.87
FBgn0250814	CG4169	3.15	2.4E–40	4.37	5.9E–32	–1.22
FBgn0263705	Myo10A	3.24	1.3E–10	4.03	2.8E–23	–0.79
FBgn0015513	mbc	3.26	3.8E–28	4.04	2.7E–150	–0.79
FBgn0026239	gukh	3.49	6.4E–44	4.17	4.6E–51	–0.68
FBgn0011225	jar	3.57	1.8E–65	4.27	1.1E–111	–0.70
FBgn0033698	CG8858	3.58	1.3E–87	4.33	1.4E–133	–0.75
FBgn0027948	msps	3.60	2.3E–77	4.24	1.0E–274	–0.65
FBgn0003464	sol	3.63	5.6E–53	4.17	1.0E–43	–0.54
FBgn0034963	Not11	3.66	2.9E–40	4.25	2.5E–41	–0.59
FBgn0031106	Syx16	3.68	2.9E–33	4.20	5.2E–31	–0.52
FBgn0032374	CG14931	3.73	2.8E–73	4.53	2.1E–81	–0.80
FBgn0034964	IntS1	3.73	2.3E–11	4.77	3.9E–99	–1.03
FBgn0262166	calypso	3.74	2.7E–23	4.11	4.1E–68	–0.37
FBgn0015277	Pi3K59F	3.76	3.9E–24	5.53	8.7E–23	–1.77
FBgn0260990	yata	3.83	4.9E–43	5.05	1.2E–67	–1.22
FBgn0005596	yem	3.86	1.9E–123	4.06	9.5E–153	–0.20
FBgn0032796	CG10188	3.88	1.9E–178	4.17	2.8E–147	–0.29
FBgn0259734	CG42388	3.89	9.0E–206	4.55	6.4E–283	–0.67
FBgn0025739	pon	3.90	2.8E–30	4.19	3.3E–24	–0.29
FBgn0030500	Ndc80	3.94	7.1E–92	4.34	5.2E–78	–0.40
FBgn0050493	CG30493	3.95	1.1E–22	4.01	3.8E–28	–0.06
FBgn0261797	Dhc64C	3.98	1.2E–134	4.01	7.6E–118	–0.04
FBgn0031321	Tgt	4.55	1.1E–55	0.42	7.5E–02	4.13
FBgn0003302	rux	4.25	1.7E–35	0.48	2.4E–02	3.78
FBgn0010097	gammaTub	4.26	1.4E–101	0.60	2.3E–02	3.66
FBgn0030938	CG15047	6.42	3.0E–48	0.61	2.5E–09	5.81
FBgn0016054	phr6-4	4.07	1.7E–129	0.99	7.0E–07	3.08
FBgn0013563	Pex1	4.37	7.2E–136	1.20	2.4E–09	3.17
FBgn0263589	CR43614	4.93	2.4E–19	1.25	2.3E–09	3.68
FBgn0029706	CG3626	5.38	9.0E–95	1.45	1.4E–17	3.93
FBgn0036770	Prestin	4.26	2.0E–149	1.60	8.6E–10	2.67
FBgn0030467	CG1764	4.04	1.3E–56	1.73	8.5E–16	2.32
FBgn0015229	glec	4.70	3.8E–156	1.73	9.3E–11	2.97
FBgn0033993	CG8089	4.22	2.5E–71	1.74	4.8E–10	2.48
FBgn0030805	wus	5.00	4.2E–150	1.82	5.4E–33	3.18
FBgn0032763	CG17568	4.17	1.4E–85	1.82	3.6E–12	2.34
FBgn0032029	CG17292	4.34	6.0E–96	1.85	1.7E–16	2.49
FBgn0030608	Lsd-2	4.57	0.0E+01	1.87	2.5E–11	2.70
FBgn0038575	CG7208	4.01	9.7E–195	1.87	1.1E–14	2.14
FBgn0050410	Rpi	4.13	3.6E–109	1.88	2.2E–22	2.26
FBgn0030740	CG9917	4.39	1.8E–248	1.88	4.4E–20	2.50
FBgn0033495		4.32	1.0E–144	1.91	2.9E–13	2.41

Supplemental data Table S3: Zygotic transcripts upregulated between preblastoderm (0–1 h) and blastoderm stage (1.5–2.5 h) in wild type embryos and embryos from *SHMT*[X238] embryos. Average of three biological replicates.

Table S3: Genes with differential zygotic induction

Genes with strong zygotic induction (N<–4) in either wild type or SHMT mutants

		wild type		SHMT		Difference
Gene Id	Name	log2	p value	log2	p value	WT–SHMT
FBgn0033108	CG15236	–7.11	2.2E–22	–2.90	6.8E–02	–4.21
FBgn0005631	robo	–6.45	6.2E–64	–3.88	9.4E–28	–2.57
FBgn0029907	Atx-1	–6.40	3.7E–27	–3.58	4.3E–06	–2.82
FBgn0263873	sick	–6.19	5.5E–18	–3.56	8.1E–09	–2.63
FBgn0002945	nkd	–6.15	2.5E–15	–3.14	2.8E–03	–3.00
FBgn0262719	CG43163	–5.73	4.5E–42	1.78	1.1E–06	–7.51
FBgn0259211	grh	–5.54	8.4E–33	–3.99	2.9E–14	–1.55
FBgn0052227	gogo	–5.39	8.5E–27	–2.83	5.4E–06	–2.57
FBgn0085412	CG34383	–5.39	5.8E–30	–3.09	7.3E–05	–2.30
FBgn0026061	Mipp1	–5.38	7.2E–19	–3.16	4.8E–10	–2.22
FBgn0002526	LanA	–5.33	2.9E–19	–3.59	9.5E–10	–1.74
FBgn0003002	opa	–5.29	8.8E–05	–2.43	1.5E–02	–2.86
FBgn0005616	Msl-2	–4.97	6.0E–50	–3.46	3.7E–23	–1.51
FBgn0001258	ImpL3	–4.91	1.1E–42	–3.37	1.0E–24	–1.53
FBgn0050431	CG30431	–4.88	2.4E–20	–3.56	3.0E–05	–1.32
FBgn0000542	ec	–4.86	1.3E–32	–3.56	4.1E–20	–1.29
FBgn0003715		–4.84	6.5E–25	–2.13	1.2E–09	–2.70
FBgn0028371	jbug	–4.82	3.5E–19	–3.36	5.7E–09	–1.46
FBgn0031717	Oscillin	–4.77	3.0E–28	–3.12	1.3E–17	–1.65
FBgn0043854	slam	–4.75	7.3E–118	–3.83	3.5E–36	–0.92
FBgn0034487	Efhc1.2	–4.69	1.3E–22	–3.68	1.9E–09	–1.01
FBgn0053555		–4.53	3.7E–49	–2.44	6.6E–18	–2.09
FBgn0004108	Nrt	–4.52	1.3E–226	–3.51	6.6E–43	–1.01
FBgn0039000		–4.51	1.2E–09	–0.33	3.1E–01	–4.19
FBgn0001234	Hsromega	–4.50	2.6E–17	–3.69	3.0E–16	–0.81
FBgn0026160	tna	–4.49	6.8E–18	–2.57	4.0E–09	–1.92
FBgn0050015	CG30015	–4.37	5.4E–34	–3.18	4.0E–49	–1.19
FBgn0000228	Bsg25D	–4.34	7.8E–231	–3.54	2.3E–81	–0.81
FBgn0015949	hrg	–4.26	5.5E–165	–3.70	2E–114	–0.56
FBgn0264089	sli	–4.05	1.9E–42	–1.43	2.9E–10	–2.62
FBgn0011706	rpr	–3.66	1.0E–16	–6.13	7.5E–17	2.47
FBgn0033483	egr	–2.94	1.9E–24	–4.37	1.0E–76	1.43
FBgn0010651	l(2)08717	–2.71	2.6E–11	–4.76	1.2E–18	2.05

Genes selected according to the difference in zygotic induction

		wild type		SHMT		Difference
Gene Id	Name	log2	p value	log2	p value	WT–SHMT
FBgn0262719	CG43163	–5.73	4.5E–42	1.78	1.1E–06	–7.51
FBgn0033108	CG15236	–7.11	2.2E–22	–2.90	0.068	–4.21
FBgn0039000		–4.51	1.2E–09	–0.33	0.39	–4.19
FBgn0031879	uif	–7.62	8.0E–19	–4.04	4.3E–08	–3.58
FBgn0000477	DNaseII	–7.49	1.E–17	–4.12	3.1E–06	–3.37
FBgn0010473	tutl	–7.86	1.2E–29	–4.57	5.8E–08	–3.29
FBgn0004878	cas	–7.80	9.9E–13	–4.57	4.0E–05	–3.23
FBgn0002945	nkd	–6.15	2.5E–15	–3.14	0.0028	–3.00
